# The first antenatal diagnosis of KBG syndrome: a microdeletion at chromosome 16q24.2q24.3 containing multiple genes including ANKRD11 associated with the disorder

**DOI:** 10.1002/ccr3.1285

**Published:** 2017-12-11

**Authors:** Victoria Hodgetts Morton, Elizabeth Quinlan‐Jones, Natasha Butts, Denise Williams, Sue Hamilton, Tamas Marton, Katie Morris

**Affiliations:** ^1^ Birmingham Women's Hospital Mindelsohn Way Birmingham B15 2TG UK; ^2^ Institute of Metabolism and Systems Research University of Birmingham Birmingham B15 2 TT UK

**Keywords:** ANKRD11, KBG syndrome, prenatal diagnosis

## Abstract

The loss of ANKRD11 gene confirms the diagnosis of KBG syndrome but does not elucidate the pediatric phenotype providing a counseling challenge. With the expansion of prenatal diagnosis, and the potential to perform whole‐exome sequencing antenatally, we must describe the genetic abnormalities, antenatal ultrasound findings, and phenotype concurrently to facilitate counseling.

## Introduction

KBG syndrome was first described by Herrmann 1 and is associated with distinct dental, craniofacial, neurobehavioral, and skeletal anomalies, macrodontia of the upper central incisors of the permanent teeth being the dominant diagnostic feature 2. Whole‐exome sequencing (WES) of affected individuals has recently identified mutations in ANKRD11 as causative of the disorder 2. KBG syndrome can occur due to various loss of function alterations in ANKRD11, or as a result of known 16q24 microdeletions encompassing ANKRD11. ANKRD11 encodes ankyrin repeat domain 11, which interacts with nuclear receptor complexes to alter transcription localizing to the nuclei of neurons, accumulating in discrete inclusions in neurons, thus supporting the role of ANKRD11 in neural plasticity 3. Diagnosis of KBG syndrome is possible in the neonatal period; however, the clinical signs can manifest mildly with few associated medical complications and as such diagnosis can be entirely missed or delayed. More often, diagnosis is made at 7–8 years of age following eruption of the upper permanent central incisors. Hence, it is suspected that KBG syndrome is an underdiagnosed condition.

## Case Report

We report the first known antenatally diagnosed case of a microdeletion at chromosome 16q24.2q24.3 encompassing ANKRD11, identified on array comparative genomic hybridization (aCGH) as causative for KBG syndrome in the fetus. We report the postmortem findings, which were confirmative of the expected phenotype associated with 16q24 microdeletion including ANKRD11, and discuss the rapidly evolving area of prenatal diagnosis in relation to the importance of appropriate counseling and support for families undergoing genetic testing.

**Figure 1:**
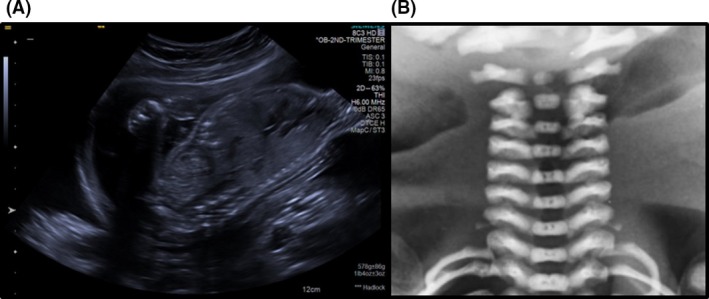
(A) Ultrasound scan image of fetus at 21 weeks showing calcified foci in the upper abdomen (liver). (B) Postmortem X‐ray of the irregularity of the upper cervical vertebrae.

A 32‐year‐old nulliparous woman was referred to a tertiary Fetal Medicine Centre at 21 weeks of gestation with suspected fetal echogenic bowel. A detailed ultrasound assessment demonstrated calcified foci in the upper abdomen (liver) (Fig. 1A). Cystic fibrosis carrier testing and infection screening were undertaken and reported as negative. An amniocentesis was performed, and chromosomal microarray (CMA) demonstrated a male fetus with a deletion of approximately 1.86 Mb at chromosome 16 band q24.2q24 3. The deleted region ISCN nomenclature for the deletion arr [GRCh37] 16q24.2q24.3 (87614996_89479537) x1 dn contained 26 genes from JPH3 including ANKRD11. A diagnosis of KBG syndrome was prospectively made. Targeted parental CMA testing demonstrated that the microdeletion occurred *de novo* in the fetus.

After counseling and consideration, the parents proceeded with termination of pregnancy at 27 + 5 weeks of gestation resulting in delivery of a male fetus weighing 950 g. Postmortem examination identified multiple external congenital anomalies including a triangularly shaped face, mildly low‐set ears, and a right retained testis. Internal congenital anomalies included incomplete lobation of the left lung, lobulated spleen, cervical ribs, irregularity of vertebral body C1‐4, and calcification of the liver associated with the portal tracts.

## Discussion

The facial and skeletal features identified at postmortem are consistent with KBG syndrome. The gastrointestinal abnormalities reported, specifically the calcification of the portal tracts in the liver, have not previously been associated with this syndrome and may therefore represent a more severe phenotype, likely due to the complete loss of ANKRD11 in addition to the loss of any of the other 25 genes located on this segment of the chromosome.

Low et al. 4 have reported the clinical and genetic aspects of KBG syndrome in 30 children following development of a single gene diagnostic test for the condition in 2014. There were no antenatal diagnoses of KBG syndrome, and on retrospective review of their prenatal anomaly scans, there was one cardiac condition identified and one single umbilical artery. Both KBG syndrome and 16q24 microdeletion syndrome have been associated with small for gestational age fetuses and polyhydramnios 4.

Antenatal diagnosis in this first reported antenatal case is secondary to performing invasive testing following identification of fetal liver calcification. There is an important association between liver calcification and chromosomal abnormalities with a 50% versus 20% significant difference in matched controls with and without antenatal identification of liver calcification 5.

The KBG diagnosis, although supported by the congenital abnormalities observed at postmortem examination, does not fully elucidate the neonatal and pediatric phenotype that might be expected and thus provides a counseling challenge.

There has been a rapid growth in the diagnosis of this condition with many suspected cases of KBG syndrome, or children with learning disabilities undergoing exome sequencing demonstrating partial gene deletions or single gene loss at ANKRD11. The case we report has loss of 1.86 Mb at chromosome 16 band q24.2q24.3. This larger deletion allowed identification through CMA rather than exome sequencing; however, there are very few cases in the literature of children and adults with a deletion of this size. Two cases are reported by Goldenberg et al. 6 with a similar loss to our case of ANKRD11 and the surrounding genes, and these children had more severe phenotypes with multiple abnormalities and learning disabilities. Since our case, a further review by Novara et al. 7 identified 12 cases with haploinsufficiency for *ANKRD11*‐flanking genes and reported a difference between KBG syndrome and 16q24.3 microdeletion syndromes which are postulated to have more severe phenotypes.

With the rapid expansion of prenatal diagnosis, and the potential to perform exome sequencing in the antenatal period, we must continue to carefully describe the genetic abnormalities, antenatal ultrasound findings, and the postnatal phenotype concurrently to facilitate the challenges in prenatal counseling and allow accurate and clear counseling to patients.

Reiff et al. 8 have previously described that parents of children undergoing CMA testing for suspected genetic anomalies struggle to understand the scientific uncertainty relating to the results they receive irrespective of whether findings are of known pathogenic contribution or are variants of uncertain significance (VOUS). This is particularly evident where the pathogenic alteration is rare or newly discovered, or if the availability of prognostic evidence is limited 8. Uncertain knowledge has been described as “toxic” by participants in a study by Berhardt et al. 9 who interviewed women at increased risk of congenital fetal anomaly following prenatal CMA testing. In retrospect, participants in this research wished they had not received uncertain information and were left feeling anxious with concerns that lingered following their child's birth 9. The stress that parents experience when opting for prenatal diagnosis may be intensified by the complex and often ambiguous results arising from the technology, thus clear and accurate verbal and written communication by health professionals is paramount. Appropriate pre and post‐test counseling can help parents understand the types of findings that may be returned to them, and is central to supporting informed decision making 10. Managing uncertainty in relation to genetic results will continue to present challenges for clinicians 10, 11 and complicate parental decisions in pregnancy 9. In time, prenatal counseling around prognosis relating to many genetic alterations will be improved as further phenotypic data are gathered leading to better parental understanding and experience 11.

## Conclusion

We present the first antenatal diagnosis of KBG syndrome, potentially demonstrating a more severe phenotype that is associated with complete loss of ANKRD11 and the surrounding genes, which includes triangularly shaped face, low‐set ears, retained testis, cervical ribs, irregularity of the vertebral bodies, incomplete lobation of the lungs, lobulated spleen, and calcification of the liver associated with the portal tracts.

## Authorship

VHM, EQJ, NB: cowrote the manuscript. DW, SH: provided clinical and laboratory input. TM: provided pathology input. RKM: provided specialist fetal medicine input and oversight.

## Conflict of Interest

None declared.
